# New Trends on Antineoplastic Therapy Research: Bullfrog (*Rana catesbeiana* Shaw) Oil Nanostructured Systems

**DOI:** 10.3390/molecules21050585

**Published:** 2016-04-30

**Authors:** Lucas Amaral-Machado, Francisco H. Xavier-Júnior, Renata Rutckeviski, Andreza R. V. Morais, Éverton N. Alencar, Teresa R. F. Dantas, Ana K. M. Cruz, Julieta Genre, Arnóbio A. da Silva-Junior, Matheus F. F. Pedrosa, Hugo A. O. Rocha, Eryvaldo S. T. Egito

**Affiliations:** 1 Disperse Systems Laboratory (LaSiD), Pharmacy Department, Federal University of Rio Grande do Norte (UFRN), Av. General Gustavo de Cordeiro-SN-Petropolis, Natal 59012-570, Brazil; machado.lucasam@gmail.com (L.A.-M.); ffhxjunior@yahoo.com.br (F.H.X.-J.); renatarut@hotmail.com (R.R.); andrezarochelle@hotmail.com (A.R.V.M.); everton_alencar@hotmail.com (E.N.A.); teresafernandes__@hotmail.com (T.R.F.D.); jgenre@gmail.com (J.G.); 2 Graduated Program in Pharmaceutical Sciences, LaSiD, UFRN, Av. General Gustavo de Cordeiro-SN-Petrópolis, Natal 59012-570, Brazil; 3 Graduated Program in Health Sciences, LaSiD, UFRN, Av. General Gustavo de Cordeiro-SN-Petrópolis, Natal 59012-570, Brazil; 4 Laboratory of Biotechnology of Natural Polymers (BIOPOL), Biochemistry Department, Federal University of Rio Grande do Norte, Av. Senador Salgado Filho-3000-Lagoa Nova, Natal 59064-741, Brazil; anakatarina1@uol.com.br (A.K.M.C.); hugo@cb.ufrn.br (H.A.O.R.); 5 Pharmaceutical Technology & Biotechnology Laboratory (TecBioFar), Pharmacy Department, Federal University of Rio Grande do Norte, Av. General Gustavo de Cordeiro-SN-Petrópolis, Natal–RN 59012-570, Brazil; arnobiosilva@gmail.com (A.A.d.S.-J.); mpedrosa@ufrnet.com (M.F.F.P.)

**Keywords:** bullfrog oil, nanomedicine, tumor cells, nanocarrier

## Abstract

Bullfrog oil is a natural product extracted from the *Rana catesbeiana* Shaw adipose tissue and used in folk medicine for the treatment of several diseases. The aim of this study was to evaluate the extraction process of bullfrog oil, to develop a suitable topical nanoemulsion and to evaluate its efficacy against melanoma cells. The oil samples were obtained by hot and organic solvent extraction processes and were characterized by titration techniques and gas chromatography mass spectrometry (GC-MS). The required hydrophile-lipophile balance and the pseudo-ternary phase diagram (PTPD) were assessed to determine the emulsification ability of the bullfrog oil. The anti-tumoral activity of the samples was assessed by 3-(4,5-dimethylthiazol-2-yl)-2,5-diphenyltetrazolium bromide (MTT) assay for normal fibroblast (3T3) and melanoma (B16F10) cell lines. Both extraction methods produced yielded around 60% and the oil was mainly composed of unsaturated compounds (around 60%). The bullfrog oil nanoemulsion obtained from PTPD presented a droplet size of about 390 nm and polydispersity = 0.05 and a zeta potential of about −25 mV. Both the bullfrog oil itself and its topical nanoemulsion did not show cytotoxicity in 3T3 linage. However, these systems showed growth inhibition in B16F10 cells. Finally, the bullfrog oil presented itself as a candidate for the development of pharmaceutical products free from cytotoxicity and effective for antineoplastic therapy.

## 1. Introduction

Ethnopharmacological exploration concerning the bio-prospection of drug discovery based on natural sources has become quite common since the isolation of new pharmacologically active compounds, increasing the therapeutic arsenal against various diseases [1,2,3,4,5]. In this context, natural oils have been widely used in the folk medicine for the treatment of several diseases due to their rich content of secondary metabolites and fatty acids [6]. These oils have a renewable character [7], low toxicity, high biodegradability, and presence of substances with pharmacological activity [8].

Bullfrog oil is a natural oil extracted through biotechnological reuse of adipose tissue, from the amphibious *Rana catesbeiana* Shaw, which is usually discarded by food industries after the bullfrog meat processing [9,10]. Previous chemical characterization of the bullfrog oil suggests a pharmacological potential due to the presence of fatty acids such as oleic, linolenic, stearic, palmitic, and myristic acids [10,11]. The oil *in-nature* has been used in folk medicine due to its inflammatory, antioxidant, antiedematogenic, and antimicrobial activities [9]. However, although there are studies showing that the presence of fatty acids can promote the viability reduction of tumor cells due to damage to the cellular membrane [12], there are no studies that evaluate this activity on bullfrog oil. Additionally, its unpleasant organoleptic characteristics, its low spread ability, its poor skin absorption, and its low viscosity can contribute to low patient adherence to the treatment [9].

Based on these advantages, the development of an emulsified system based on bullfrog oil would become a simple and effective alternative to its use. Emulsified systems are thermodynamically unstable dispersions, composed of a mixture of two or more immiscible liquids stabilized by surfactants. According to the physicochemical properties of a specific oil, a suitable surfactant mixture can efficiently stabilize the droplets into an aqueous solution. The composition of this mixture is assessed by the evaluation of its hydrophile-lipophile balance (HLB) [13,14], which is an empirical number proposed by Griffin [15] that affects the stability and the type of emulsion systems [15,16,17].

The aim of this study was to develop a stable nanoemulsion containing bullfrog oil for topical application. Initially, the oil was extracted and characterized, followed by HLB study. A pseudo-ternary phase diagram was plotted according to the visual aspect of different samples prepared by mixing the oil, water, and surfactants, and a point was chosen to produce the bullfrog oil nanoemulsion system. In addition, the *in vitro* biocompatibility and cytotoxicity were assessed by the 3-(4,5-dimethylthiazol-2-yl)-2,5-diphenyltetrazolium bromide (MTT) assay for normal fibroblasts (3T3) and melanoma (B16F10) cell lines, respectively.

## 2. Results and Discussion

### 2.1. Extraction and Physicochemical Characterization of the Bullfrog Oil

The extraction of bullfrog oil was performed following two different methods in order to produce the BOH (bullfrog oil extracted by hot method) and BOHx (bullfrog oil extracted using n-hexane as organic solvent). The yields of bullfrog oil extractions were 60.6% and 74.8% concerning the fatty tissue used in the extraction processes for hot and solvent extractions, respectively. Differences in the yield of oil extraction can be obtained using various techniques in association with changes in temperature, solvents, and pressure [18]. Lopes and colleagues [10] evaluated bullfrog oil extraction yield under different temperatures and found that the income is directly proportional to the extraction temperature, which corroborates our findings.

**Figure 1:**
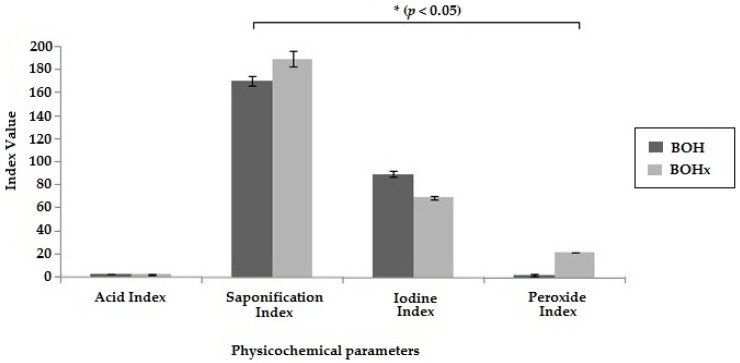
Physicochemical parameters resulting from the analysis of bullfrog oil. Acid index (mg of KOH/g of oil); Saponification index (mg of KOH/g of oil), Iodine index (g of iodine absorbed/100 g of oil), and Peroxide index (mEq of active oxygen/1000 g of oil). Dark gray: bullfrog oil extracted by heating process (BOH); Light gray: bullfrog oil extracted by the organic solvent process (BOHx). * Statistical Difference.

The quality of the oils extracted was evaluated by following their physicochemical characterization. These parameters were important because they can be used to evaluate the quality of the bullfrog oil extracted, since the standards of the bullfrog oil were not available in the literature [19]. The acid index (AI) was used to determine the hydrolytic degradation of the bullfrog oil, as high AI suggests the presence of water [19]. BOH and BOHx showed the same AI (2.9 mg of KOH/g of oil) (Figure 1), demonstrating good preservation of bullfrog oil after the extraction process. The iodine index (II) was performed to evaluate the degree of unsaturation of the fatty acids in the oil, allowing analysis of its purity [20]. Both BOH and BOHx demonstrated a high degree of fatty acid unsaturation; however, BOH showed higher concentration of iodine (Figure 1) than BOHx. These results were similar to those found in the literature [21], indicating that the bullfrog oil has high amounts of unsaturated fatty acids. Therefore, this finding suggests that the presence of the organic solvent in the bullfrog oil extraction process can promote a greater extraction of saturated fatty acids. The extraction with organic solvent is based on the compounds’ polarity and on the increasing surface contact between the adipose tissue and the organic solvent, while the heating extraction method is based on the lipid melting temperature. Additionally, this explains the greater yield in the extraction using organic solvent, as the chosen method affects the extraction performance [22].

The saponification index (SI) was evaluated to determine the relative quantity of saturated fatty acids. BOHx had a higher SI when compared to BOH, corroborating the II result, since the long chain fatty acids have a low SI. The peroxide index (PI) is a parameter analysis for oxidative degradation, as peroxide is the first compound formed during the oxidation process [23]. The results obtained for PI (Figure 1) indicate that the organic solvent extraction process provided greater PI than the hot extraction. Choe and Min [24] suggested that PI is directly proportional to the amount of free fatty acids. Thus, it can be inferred that BOHx may have a greater number of free fatty acids, promoting the acceleration of the oxidation process. Therefore, these results suggest that the hot extraction method allows the production of a pure oil with an increased amount of unsaturated fatty acids, free of organic solvent traces and lower levels of peroxide, constituting the best extraction method for the bullfrog oil.

Table 1 shows the chemical composition of the main compounds identified in the BOH using GC-MS. The major unsaturated compounds found were the eicosapentaenoic acid (EPA) (17.6%) and the arachidonic acid (8.4%), while the major monounsaturated compound was the oleic acid (29.9%). Studies about the chemical characterization of the bullfrog oil showed that the unsaturated fatty acids are present at lower concentrations (26.8%) than the saturated fatty acids (53.6%). Additionally, it was observed that these unsaturated fatty acids belong to the omega family [9,10,11,25]. Although these studies had identified the same compounds, it was possible to observe changes in their concentrations. Indeed, in this study the arachidonic acid concentration was 8.4%, while Silva and colleagues [25] and Lopes and colleagues [10] identified the same compound in the bullfrog oil at 0.74% and 0.6%, respectively. Significant differences were observed for EPA (17.6%) and docosahexaenoic acid (DHA) (0.8%) in comparison to Silva (0.46% and 0.91%) and Lopes (0% to 0.1%) studies. These differences may be related to both the feeding of these amphibians and the climatic conditions of the frog farms. The results obtained are relevant, considering that the arachidonic acid acts as a proinflammatory agent stimulating the synthesis of leukotrienes and prostaglandins, promoting leukocyte migration and accelerating the onset of the first phase of the skin healing process [26,27,28,29]. EPA and DHA play important roles in the human body, such as preventing cardiovascular disease and improving mental capacity [30], besides providing greater permeability of drugs in topical treatments [31].

Ethyl iso-allocholate was also identified in the BOH sample, even though it was not described in previous studies. This bioactive compound presents antioxidant, antibacterial, anti-tumor, cancer preventive, diuretic, anti-inflammatory, and anti-asthma properties [32,33].

The presence of all these compounds with therapeutic activities in the bullfrog oil makes it especially suitable for topical use, mainly by its compound interaction with ceramides, reducing skin dehydration [11,25,34]. Therefore, the bullfrog oil could present several therapeutic applications in the context of different diseases, with great pharmacological potential, mainly considering its low cost production through biotechnological processes.

### 2.2. Pseudo-Ternary Phase Diagram (PTPD) for Bullfrog Oil Compositions

Table 2 shows the physicochemical properties of different bullfrog oil nanoemulsions prepared by using a surfactant blend composed of Tween^®^ 20 and Span^®^ 80 at distinct weight:weight (*w*/*w*) ratios (HLBr from 4.5 to 15.5). HLB is an important parameter to produce stable emulsified systems, since the mixtures must interact better with a specific oil phase [35]. In the first batch, it was possible to observe that nanoemulsions with HLB between 12.5 and 13.5 showed the smallest droplet size variations. No significant differences between the systems at HLB 12.5 and 13.5 were established, due to the small droplet size variation (259.9 nm up to 275.6 nm) and the lowest polydispersity (PDI) (0.190 up to 0.218). This interval was considered the most capable of producing stable nanoemulsion systems. In addition, the lowest creaming rates were also observed for the same interval (2.8% at 3.7%). All samples showed an acid pH, which is the result of the large number of long chain fatty acids presented in the chemical composition of the bullfrog oil.

In a second step, this HLB range was investigated by using smaller variations (about 0.1) with an interval of confidence corresponding to half of the variation of the HLB range used in the first batch. Table 3 shows the results obtained for the characterization of the nanoemulsion systems for the second batch (HLBr ranged from 12.0 to 14.0) after 60 days at 25 °C. The higher creaming rate observed in the bullfrog nanoemulsion was 3.2% (±0.8) for the formulation with the HLB 13.6 (Table S1). The pH remained weakly acid during all the studied time, ranging from 5.0 (±1.6) to 5.6 (±1.4). The pH value is an important factor in the stability of the emulsified systems, particularly when the emulsifier agents are obtained by the saponification process, since the acid compounds may promote destabilization of the emulsifier system leading to phase separation [36]. Furthermore, in cases in which some skin damage exists due to the internal alkalinity of the lesions, the acid pH protects the skin and can also accelerate the healing process [37,38]. In this study, the pH decreased progressively over the time (1–60 days). These results suggest a microbial contamination or degradation of the fatty acids with a long carbon chain presented in the bullfrog oil, which can be oxidized to form hydroperoxides or hydrolysis of triglycerides [11,39,40,41]. Regarding the electrical conductivity, the high value found allowed us to speculate that the oil was dispersed in the water phase, producing an oil-in-water (O/W) nanoemulsion [42,43].

The selected surfactant (Tween^®^ 20 and Span^®^ 80) blend was efficient to stabilize the bullfrog oil into water, once the second batch of the nanoemulsions presented droplet sizes between 188 and 220 nm (*p* > 0.05). In addition, no flocculation or coalescence was visually identified, since these phenomena are governed by the Ostwald ripening [44,45]. These results suggest that the bullfrog oil has an HLB range from 12.0 to 13.5. However, the system that showed the lowest variation in the droplet size and the lowest PDI throughout the study was the system produced with a HLB 12.1. Thus, the required HLB (HLBr) for bullfrog oils was established as 12.1. At this HLB, the optimal concentration of the formulation compounds was also established, and the pseudo-ternary phase diagram was constructed using this HLBr as a fixed variant.

**Figure 2:**
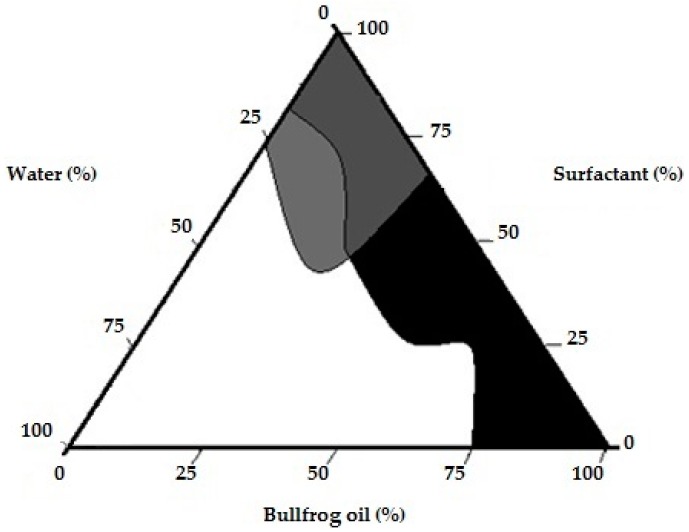
Pseudo-ternary phase diagram of the bullfrog oil produced with **HLBr** 12.1. Black (phase separation), dark gray (microemulsion), medium gray (gel), white (emulsion).

The pseudo-ternary diagram is a useful tool in the development of dispersed systems since it is possible to obtain the optimal compositions able to produce the required system [46,47,48,49,50,51]. Different emulsified systems such as emulsion, microemulsion and nanoemulsion, can be characterized by this method. These systems differ in their thermodynamic stability and physicochemical properties [45,52]. Indeed, microemulsion is defined as a thermodynamically stable system, whereas emulsion and nanoemulsion are considered unstable. However, nanoemulsions, which present a smaller droplet size (around 300 nm) than emulsions, presented a better kinetic stability [53]. A large region of emulsified systems was obtained during the pseudo-ternary construction (68%) (Figure 2). Different emulsion systems with water ratio ranging from 20% to 80% or with oil ratio up to 60% were observed. Similar results were observed for emulsions prepared with mono-, di- and triglyceride of medium chain fatty acids as oil phase (30% to 50%) [51] and Espinosa and colleagues [47] showed the formation of the emulsion with lipid concentrations ranging from 5% to 70%. The presence of clear flow-resistant dispersions (gels, 2%) and clear and transparent liquid dispersions (microemulsions, 4%) were also observed. The pseudo-ternary phase diagram was successfully used in similar studies as a tool to determine the best concentration of components for emulsified systems [48,49,50]. Therefore, in order to guarantee a suitable topical nanoemulsion, the oil-in-water (O/W) basic nanoemulsion formulation containing 12% of bullfrog oil, 8% of surfactant blend (Tween^®^ 20:Span^®^ 80 6.29:3.71 ratio), and 80% of distilled water was chosen for further studies.

### 2.3. Development of a Topical Bullfrog Oil Nanoemulsion

Some additives were added to the chosen nanoemulsion point on the PTPD studies in order to improve the organoleptic characteristics of the bullfrog oil. The effect of these substances on the stability of the bullfrog oil nanoemulsion was evaluated for 90 days. The basic nanoemulsion sample (without additives) was used as a control.

Macroscopic aspects such as white color and absence of the characteristic odor of bullfrog oil remained in the samples throughout the studied time. The basic nanoemulsion presented himself as a fluid system; however, this characteristic was changed in the topical nanoemulsion due to the addition of xanthan gum at 1%, which generated a high viscosity system. The creaming rate value of about 2.4% was observed for the basic nanoemulsion. A similar result (about 2.5%) was observed in the HLBr study. Nevertheless, the topical nanoemulsion did not show creaming at all, which probably occurred due to the high viscosity found in these systems. This feature reduces the motility of the droplets, improving the emulsion stability [54]. The creaming rate is an important parameter to evaluate the stability of the emulsion systems, since it precedes the phase separation phenomena.

**Figure 3:**
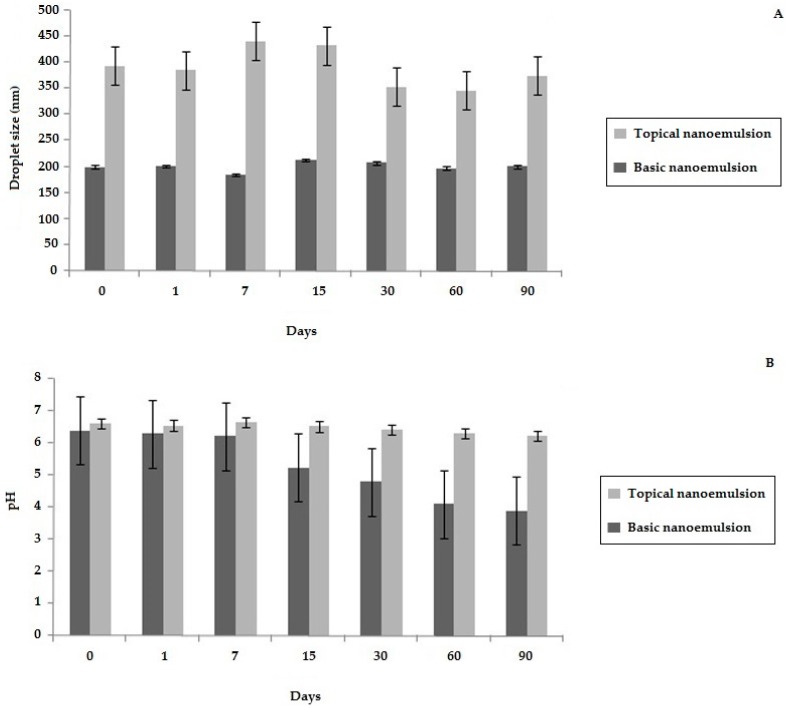
Droplet size and pH analyses for the basic emulsion and the topical nanoemulsion for 90 days. (**A**) droplet size analyses; (**B**) pH analyses. Dark gray: basic nanoemulsion; Light gray: topical nanoemulsion.

Figure 3 shows the results of droplet size and pH for the two different samples, in which a significant increase in the droplet size occurred for the topical nanoemulsion after the incorporation of the additives. However, the droplet size remained constant during the entire time of the study (Figure S1) confirming the absence of the flocculation or coalescence phenomenon for both samples. In addition, the zeta potential of the basic nanoemulsion (−32.79 mV ± 2.90) and the topical nanoemulsion (−25.02 mV ± 4.33) suggested that these systems were quite stable. The values of zeta potential above 25 mV (absolute values) indicate that the repulsive forces are greater than the attractive ones, keeping the droplets dispersed [55,56,57]. These results demonstrated the importance of the selected surfactant pair (Tween^®^ 20:Span^®^ 80 at the ratio of 6.29:3.71) for the stability of bullfrog oil nanoemulsions. Furthermore, the selected additives incorporated in the topical nanoemulsion avoided the pH variation observed for the basic nanoemulsion. The absence of antimicrobial preservatives and antioxidants in the basic nanoemulsion may be suggestive of microbiological or chemical degradations, changing the smell, color, and pH values of the formulation [58].

The stability study also evaluated the organoleptic characteristics, centrifuge resistance, and freeze/thaw cycles. Neither sample showed changes in either the color or the odor. The centrifuge resistance immediately after system preparations did not demonstrate changes in either sample, predicting suitable physical stability of the different emulsions [42]. The freeze/thaw cycle study revealed that the basic nanoemulsion presented phase separation after the second cycle, while the topical nanoemulsion remained stable until the sixth cycle, indicating that the addition of excipients in the topical formulation enhanced its stability under large temperature variations [59].

### 2.4. Biocompatibility and Cytotoxicity Study

**Figure 4:**
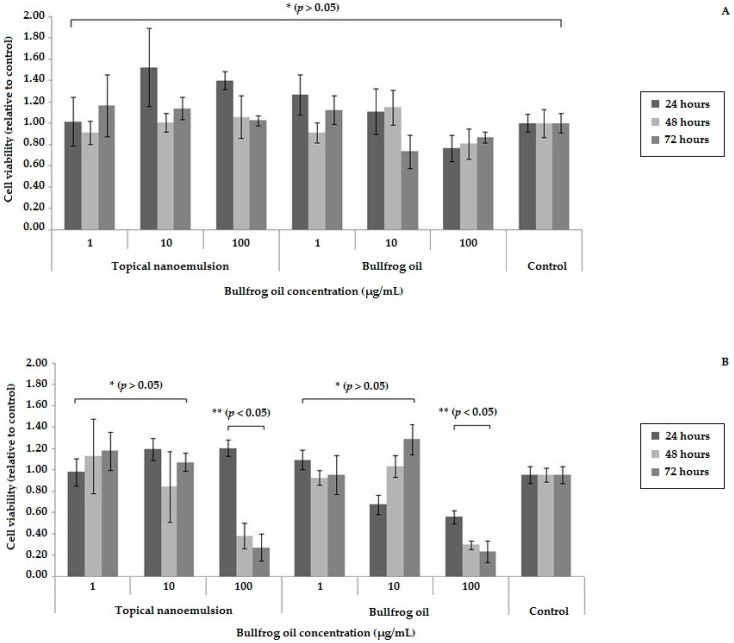
Cell viability of the topical nanoemulsion and the bullfrog oil at three concentrations. (**A**) 3T3 cell viability; (**B**) B16F10 cell viability. Dark gray: 24 h; Light gray: 48 h; Medium gray: 72 h. * No statistical difference compared to the control. ** Statistical difference compared to the control.

The biocompatibility of the 3T3 and the cytotoxicity of B16F10 cells treated with bullfrog oil or topical nanoemulsion at three different concentrations for 24 h, 48 h, and 72 h, respectively, was provided by the colorimetric MTT assay (Figure 4).

Compared to the positive control, 3T3 cell growth showed no statistical difference when treated with bullfrog oil or topical nanoemulsion samples (*p* > 0.05). These results were observed for all samples over 72 h (Figure 4A). Therefore, both samples presented biocompatibility at these concentrations for 3T3 cells, suggesting that the bullfrog oil may be suitable to be applied into a formulation. This is an important result because the safety knowledge of the natural products is very important for food and pharmaceutical industry application [3,4,9].

Similarly, results obtained for the melanoma cell line B16F10 showed that the lower concentration of both bullfrog oil and topical nanoemulsion (1 µg/mL and 10 µg/mL) did not interfere with the cell viability. However, at higher concentrations (100 µg/mL), the viability of B16F10 cells was reduced over the time. In the first 24 h, it was observed that the bullfrog oil promoted a greater inhibition of cell growth compared to topical nanoemulsion, and this occurs because the bullfrog oil is internalized in the droplet, requiring more time to be delivered to the cellular membrane. However, cell growth inhibition was not significantly different after 48 h of cell culture. Both the topical nanoemulsion and the bullfrog oil showed concentration and time-dependent cytotoxicity against B16F10 cell lineage. Similar results were obtained by our team with other tumor cell lines (cervical tumor cells—(HeLa) and liver tumor cells—(HepG2)) [60].

This potential antineoplastic activity of the topical nanoemulsion may be related to the presence of ethyl iso-allocholate and fatty acids, which were identified in the bullfrog oil. Indeed, some studies have reported the anti-tumor activity of these compounds [12,32].

Based on these results, it is possible to suggest that the bullfrog oil is a safe natural oil compound. Therefore, using it *in natura* form or adding it to a nanoemulsion system for topical use would be suitable, as it shows no cytotoxicity. However, when incorporated in a nanoemulsion system, an improvement in the organoleptic characteristics of the bullfrog oil was observed. Additionally, the bullfrog oil and the topical nanoemulsion showed inhibitory activity on the cellular growth of B16F10 cells, suggesting that this system would be a potential anti-melanoma agent. However, further *in vitro* studies with other normal and melanoma cell lines need to be conducted to confirm this hypothesis.

## 4. Materials and Methods

### 4.1. Materials

Bullfrog adipose tissue was kindly provided by the Asmarana Produtos Naturais (Natal, RN, Brazil). Span^®^ 80 (sorbitan monooleate 80), penicillin-streptomycin, and MTT reagent ((3-(4,5-dimethylthiazolyl-2)-2,5-diphenyltetrazolium bromide)) were purchased from Sigma Aldrich Inc. (St. Louis, MO, USA). Ethanol, dimethylsulfoxide (DMSO), n-hexane, propylene glycol, and Tween^®^ 20 (polyoxyethylene 20 sorbitan monolaurate) were acquired from VETEC (Rio de Janeiro, RJ, Brazil). Cetostearyl alcohol ethoxylate and isopropyl palmitate were purchased from ViaFarma (São Paulo, SP, Brazil). Xanthan Gum and Germall^®^ were purchased from Mapric (São Paulo, SP, Brazil). Butylhydroxytoluene was acquired from Galena (Campinas, SP, Brazil). Floral green fragrance was obtained from Bio Inter (São Paulo, SP, Brazil).

Dulbecco’s Modified Eagle Medium (DMEM) and fetal bovine serum (FBS) were purchased from Cultilab (Campinas, SP, Brazil). The 3T3 fibroblasts and B16F10 cell lines from the American Type Culture Collection (ATCC) used in this study were provided by the Laboratory of Biotechnology of Natural Polymers at Federal University of Rio Grande do Norte (Natal, RN, Brazil).

### 4.2. Methods

#### 4.2.1. Bullfrog Oil Extraction

Two bullfrog oil samples were extracted from the amphibian’s adipose tissue by two different methods. The first bullfrog oil sample (BOH) was produced following the method adapted from Lopes and colleagues [10]. The adipose tissue was heated under controlled magnetic stirring (IKA^®^, RH basic model KT/C, Staufen, Germany) at 80 °C for 40 min. The second bullfrog oil sample (BOHx) was obtained by the solvent-extraction technique by adding n-hexane in the adipose tissue followed by Ultra Turrax^®^ T-18 stirring at 5000 r.p.m (IKA^®^, Staufen, Germany). Then, the solvent was evaporated in a rotary evaporator (Quimis Diadema, SP, Brazil) for 30 min at 40 °C. After the extraction process, both bullfrog oil samples were filtrated in membranes of 0.45 µm (Merck Millipore, Hessen, Germany) and stored in amber glass bottles at room temperature.

#### 4.2.2. Physicochemical Characterization of Bullfrog Oils

Physicochemical analyses of bullfrog oil samples (BOH and BOHx) were carried out by titration according to the methodologies described in the United States Pharmacopeia (USP 35) [61] for the iodine index (II) and in the American Oil Chemists Society guidelines [19] for the saponification index (SI), acid index (AI), and peroxide index (PI). The II was evaluated by titration of the samples (200 mg), 10 mL of chloroform, and 25 mL of iodine bromide. After 30 min of resting, 30 mL of potassium iodide at 10% and 100 mL of water were added and titrated with sodium thiosulfate (0.1 N), using starch as an indicator. The SI was determined by titration of 400 mg of bullfrog oil mixed with potassium hydroxide (0.5 N) with hydrochloric acid (0.5 N), using alcoholic phenolphthalein as an indicator. The AI was assessed by titration of 500 mg of bullfrog oil mixed with 2.5 mL of ether:alcohol (1:1 *v*/*v*). The sodium hydroxide (0.1 N) was the titration solution and phenolphthalein was used as an indicator. PI was determined using 500 mg of bullfrog oil dissolved in 3 mL of acetic acid:chloroform (3:2 *v*/*v*). After complete dissolution, 0.05 mL of potassium iodide and 3 mL of water were added and the mixture was titrated with sodium thiosulphate (0.01 N) using starch as an indicator.

#### 4.2.3. Gas-Chromatography—Mass Spectroscopy Analysis

The identification of bullfrog oil compounds was performed using a Gas-Chromatographer equipped with an ITQ Tune mass selective detector (GC-MS) (Thermo Scientific, Waltham, MA, USA). A fused silica capillary column (25 m × 0.32 mm i.d., 0.5 µm thickness) film coated with cross-linked 5% Phenyl Polysilphenylene-siloxane (SGE Analytical Science Pty Ltd., Victoria, Melbourne, Australia) was used. GC–MS injector was set at 250 °C and the column set at 90 °C, with heating ramp from 2 °C·min^−1^ to 150 °C and then isothermally heating from 20 °C·min^−1^ to 300 °C. The split ratio was 1:25 and the electron ionization system was set at 70 eV. Helium was used as carrier gas at 1 mL·min^−1^. Samples were derivatizated with *N*,*O*-bis(trimethylsilyl)trifluoroacetamide (BSTFA) reagent and the injection volume was 1 µL. The oil components were identified by comparing their mass fragmentation with the data from the electronic library (MAINLIB, WILEY 6, TOX.HP, Acides.hp, SAMM, arp_cnrs.HP, RTLPEST3.HP, and pmw.tox2) and the data published elsewhere.

#### 4.2.4. HLBr Study

The required hydrophile-lipophile balance (HLBr) of bullfrog oil was determined based on the development of systems containing 92% of distilled water, 5% of bullfrog oil, and 3% of different ratios of surfactant blends (Tween^®^ 20 and Span^®^ 80). The nanoemulsions were developed by the phase inversion technique [62], in which aqueous and oil phases were heated separately at 70 °C. Posteriorly, the aqueous phase was added into the oily phase under constant stirring at 11,000 r.p.m by Ultra-Turrax^®^ T-18 (IKA, Staufen, Germany) for 10 min. Finally, all samples were placed in test tubes at 25 °C and 45 °C for posterior analysis. The first batch of nanoemulsions were produced with a surfactant blend in which the HLB ranged from 4.5 to 15.5, with intervals of 1.0, resulting in 13 systems. The samples that showed a lower variation of physicochemical proprieties during analyses for 60 days were chosen to develop the second batch. For this second batch, the HLB interval was 0.1 and the new systems were also physicochemically analyzed for 60 days.

#### 4.2.5. Construction of Pseudo-Ternary Phase Diagram

The pseudo-ternary phase diagram based on the water titration method at room temperature was developed to produce dispersed systems with different component concentrations [63,64,65]. Tween^®^ 20 and Span^®^ 80 were mixed at 6.29:3.71 ratios. Thereafter, the bullfrog oil was mixed with the surfactant blend at ratios from 1:9 to 9:1 and titrated with distilled water. Thereby, 90 different formulations were produced using Ultra Turrax^®^-T-18 stirring at 11,000 r.p.m for 10 min each. The systems were characterized by visual inspection in order to identify nanoemulsions, which is optically translucent and scatter light due to the Tyndall effect [42,53], microemulsion in which the system presented transparent appearance [45,52] and emulsion systems that showed a milky aspect [9,17]. Gels were defined as systems that showed clear visual appearance and high viscosity. Finally, phase separation was defined as systems with macroscopic oil droplets on the surface.

#### 4.2.6. Development of Topical Nanoemulsion Based on Bullfrog Oil

A basic nanoemulsion was defined as an O/W-emulsified system containing high oil concentration and low surfactant concentration without additives obtained from the pseudo-ternary phase diagram. In order to improve the organoleptic properties and the stability of the systems containing bullfrog oil, a topical nanoemulsion with additives was produced (Table 4). The basic and topical nanoemulsions were prepared by the phase inversion technique, as previously described in Section 4.2.4.

### 4.3. Characterization of the Emulsions

Characterization studies were performed for basic and topical nanoemulsions containing bullfrog oil.

#### 4.3.1. Macroscopic Aspect

Organoleptic changes (color and odor), as well as the presence of emulsion instability phenomena (creaming or phase separation) were determined by visual observation of the samples stored in scintillation vials at 25 and 45 ± 2 °C. The storage time for the HLB and stability studies was 60 and 90 days, respectively.

#### 4.3.2. pH and Conductivity Evaluation

The pH and the electrical conductivity of the nanoemulsions were evaluated in triplicate by pH-meter (Tecnal, TEC-2, Piracicaba, SP, Brazil) and conductometer (Digimed, DM-32, São Paulo, SP, Brazil) pre-calibrated at 25 ± 2 °C, respectively.

#### 4.3.3. Droplet Size Distribution and Zeta Potential Analysis

The hydrodynamic diameter and the size distribution of the nanoemulsions were determined in triplicate by dynamic light scattering using a ZetaPlus (Brookhaven instruments, Holtsville, NY, USA) apparatus at 25 °C. The scattered angle was fixed at 90°. Samples were diluted at 1:100 with distilled water before analysis. Results were expressed as the mean hydrodynamic diameter, the standard deviation of the size distribution, and the polydispersity index (PDI). The zeta potential of the nanoemulsions was measured by Laser Doppler Electrophoresis using a ZetaPlus (Brookhaven instruments, Holtsville, NY, USA) apparatus. To maintain a constant ionic strength, samples were diluted (1:100) in saline solution (NaCl) at 1 mM. All results corresponded to the average of three determinations.

### 4.4. Stability Study of the Emulsion

A stability study for over 90 days for the basic and the topical nanoemulsions containing bullfrog oil was performed by using three different approaches.

First, the micro-emultocrit technique (short-term stability) [17] was assessed to evaluate the creaming rate presented by the emulsions in a micro-centrifuge (Microspin, SPIN 1000 model, Equipar, PR, Brazil) at 11,500 r.p.m for 10 min. Second, the freeze/thaw cycle stability was performed by methodology adapted from Roland and Xavier [42,57] when the emulsified systems were added in hermetically sealed tubes, vertically stored for 24 h in a freezer at −5 °C, followed by another period of 24 h at 45 ± 2 °C. Cycles were repeated six times and the changes were recorded. Finally, the stability under centrifugation was determined by using 10 mL of the emulsion, placed in the centrifuge at 3000 *g* for 30 min at 25 °C.

### 4.5. Biocompatibility and Cytotoxicity Study

Two representative cell lines of skin tissue, fibroblasts (3T3) and melanoma (B16F10) lineages, were used for the biocompatibility and cytotoxicity evaluation, respectively. The assay was performed in four replicates for each cellular line and at three different concentrations of bullfrog oil or nanoemulsion (1 µg/mL, 10 µg/mL and 100 µg/mL). To attain such concentrations, the bullfrog oil was firstly diluted to 1% (*v*/*v*) with DMSO, and, then, both the bullfrog oil DMSO solution and the topical nanoemulsion (amount corresponding to the same bullfrog oil concentration) were diluted with DMEM medium. DMEM medium was also used as the positive control. Then, 100 µL of cells in DMEM medium supplemented with 10% of fetal bovine serum were placed into 96-well plates (5 × 10^4^ cells/well) and incubated overnight at 37 °C and 5% CO_2_ for a period of 24, 48, and 72 h in the presence of the aforementioned concentrations of topical nanoemulsion and bullfrog oil. Posteriorly, 100 µL of MTT reagent at 1 mg/mL was added to each well to analyze the cell viability. After 4 h, formazan crystals were dissolved in 100 µL of ethanol and the absorbance was measured in a Multiskan Ascent Microplate Reader (Thermo Labsystems, Franklin, MA, USA) at 570 nm. The biocompatibility and cytotoxicity was evaluated by the relative absorbance value between the control, the bullfrog oil, and the nanoemulsion system.

### 4.6. Statistical Analyses

All results were performed in triplicate and expressed as mean ± standard deviation. Statistical significance among three or more groups was evaluated by the variance analysis (ANOVA) followed by the Tukey test for multiple means comparisons. Analyses between the two groups were performed by the Student’s *t*-test. *p* < 0.05 was considered statistically significant.

## 5. Conclusions

The bullfrog oil, which showed a rich composition of unsaturated fat acids such as eicosapentaenoic acid (17.6%) and arachidonic acid (8.4%), was obtained by hot extraction from the adipose tissue of *Rana catesbeiana* Shaw. The HLB 12.1 produced a stable bullfrog oil emulsion. The basic nanoemulsion developed in this work was composed of Span^®^ 80 (2.97%), Tween^®^ 20 (5.03%), bullfrog oil (12%), and water (80%) (*w*/*w*). The selected additives improved the organoleptic characteristics of the bullfrog oil nanoemulsion for topical application, allowing the development of a stable nanoemulsion. The bullfrog oil and topical nanoemulsion showed biocompatibility in the normal fibroblast lineage. In addition, the bullfrog oil and the topical nanoemulsion showed a cytotoxicity activity over the melanoma cell lines (B16F10). Thus, this discovery may contribute to the design of new drugs for cancer therapy through the use of a natural product at low cost and with no toxicity.

## Figures and Tables

**Table 1:** Chemical characterization by GC-MS of the bullfrog oil obtained by hot extraction (BOH).

Substance	Retention Time (min)	Concentration (%)
Myristic acid	10.3	1.4
Arachidonic acid	12.0	8.4
Palmitic acid	12.2	10.3
EPA, Timnodonic acid	13.7	17.6
Oleic acid	13.7	29.9
Stearic acid	14	2.5
DHA, Cervonic acid	16.5	0.8
Cholesterol	20.6	9.5
Ethyl iso-allocholate	27.7	3.5
Total		83.9
Not identified		16.1

GC-MS (gas chromatography mass spectrometry); EPA (eicosapentaenoic acid); DHA (docosahexaenoic acid).

**Table 2:** Characterization of the first batch of nanoemulsion systems based on bullfrog oil to determine the HLBr after 60 days of evaluation at 25 °C.

HLB	Droplet Size (nm) ± SD	Polydispersity	Micro-Emultocrit (%) ± SD	pH ± SD	Conductivity (S/cm)
4.5	PS	PS	PS	PS	PS
5.5	PS	PS	PS	PS	PS
6.5	PS	PS	PS	PS	PS
7.5	PS	PS	PS	PS	PS
8.5	266.7 ± 14.2	0.235	4.1 ± 1.3	4.2 ± 0.6	94.4
9.5	257.7 ± 13.7	0.261	3.6 ± 1.2	3.9 ± 0.5	121.2
10.5	295.9 ± 24.8	0.268	4.3 ± 1.5	4.9 ± 1.0	107.7
11.5	PS	PS	PS	PS	PS
12.5	259.8 ± 8.7	0.218	3.0 ± 0.6	4.2 ± 0.6	100.3
13.5	275.6 ± 11.9	0.190	2.8 ± 0.7	3.8 ± 0.6	175.7
14.5	324.3 ± 26.0	0.230	3.6 ± 0.8	4.0 ± 0.4	101.4
15.5	PS	PS	PS	PS	PS

HLBr (required hydrophile-lipophile balance); SD (standard deviation); PS (phase separation).

**Table 3:** Characterization of the second batch of nanoemulsion systems based on bullfrog oil to determine the HLBr after 60 days of evaluation at 25 °C.

HLB	Droplet Size (nm) ± SD	Polydispersity	Micro-Emultocrit (%) ± SD	pH ± SD	Conductivity (S/cm)
12.1	212.0 ± 13.6	0.213	2.4 ± 0.5	5.2 ± 1.4	91.2
12.2	202.7 ± 21.6	0.22	2.4 ± 0.5	5.1 ± 1.5	100.8
12.5	202.9 ± 29.8	0.215	2.2 ± 0.7	5.4 ± 1.2	87.3
12.7	199.6 ± 16.6	0.232	2.7 ± 0.4	5.1 ± 1.5	108.7
12.9	209.3 ± 18.4	0.234	2.1 ± 0.3	5.3 ± 1.4	97.4
13.0	196.4 ± 17.3	0.215	2.0 ± 0.0	5.2 ± 1.4	90.5
13.3	201.9 ± 15.0	0.215	2.5 ± 0.5	5.0 ± 1.5	100.1
13.5	208.1 ± 19.9	0.245	2.4 ± 0.5	5.1 ± 1.4	120.5
13.7	194.4 ± 11.7	0.225	2.4 ± 0.5	5.0 ± 1.5	72.5
13.9	193.7 ± 18.5	0.248	2.5 ± 0.5	5.0 ± 1.5	112.0
14.0	188.4 ± 22.2	0.261	2.7 ± 0.5	5.0 ± 1.5	120.4

HLBr (required hydrophile-lipophile balance); SD (standard deviation).

**Table 4:** Composition of the topical nanoemulsion.

	Excipients	% (*w*/*w*)	Function
**Aqueous phase**	**Tween^®^ 20**	5.03	Surfactant
	**Propylene glycol**	4.00	Humectant
	**Germall**	0.30	Antimicrobial preservative
	**Xanthan gum**	1.00	Stabilizing agent
	**Distillated Water**	62.55	Dispenser agent
**Oily phase**	**Span^®^ 80**	2.97	Surfactant
	**Butylhydroxytoluene**	0.10	Antioxidant
	**Cetostearyl alcohol ethoxylate**	8.00	Viscosity-increasing agent
	**Isopropyl palmitate**	4.00	Emollient and skin penetrant
	**Bullfrog oil**	12.00	Oil
**After preparation**	**Fragrance**	0.05	Essence

## Supplementary Materials

Supplementary materials can be accessed at: www.mdpi.com/1420-3049/21/5/585/s1.
