# Supplementary material for: New Trends on Antineoplastic Therapy Research: Bullfrog (Rana catesbeiana Shaw) Oil Nanostructured Systems
Source: Molecules. 2016 Apr 30;21(5):585. doi: 10.3390/molecules21050585 (PMC6273763; doi:10.3390/molecules21050585)
Supplement: Supplementary file 1 [file molecules-21-00585-s001.pdf]

Supplementary Materials: New Trends on Antineoplastic Therapy Research: Bullfrog (*Rana catesbeiana* Shaw) Oil Nanostructured Systems

Lucas Amaral-Machado, Francisco H. Xavier-Júnior, Renata Rutckeviski, Andreza R.V. Morais, Éverton N. Alencar, Teresa R.F. Dantas, Ana K. M. Cruz, Julieta Genre, Arnóbio A. da Silva-Junior, Matheus F.F. Pedrosa, Hugo A.O. Rocha and Eryvaldo S.T. Egito

Table S1. Characterization of the second batch of nanoemulsion systems based on bullfrog oil to determine the HLBr after 60 days of evaluation at 25 °C.

HLB	Droplet Size (nm) ± SD	Polydispersity	Micro-Emultocrit (%) ± SD	pH ± SD	Conductivity (S/cm)
12.0	203.5 ± 20.6	0.23	2.4 ± 0.7	5.6 ± 1.4	105.6
12.1	212.0 ± 13.6	0.213	2.4 ± 0.5	5.2 ± 1.4	91.2
12.2	202.7 ± 21.6	0.22	2.4 ± 0.5	5.1 ± 1.5	100.8
12.3	205.6 ± 21.7	0.242	2.2 ± 0.4	5.3 ± 1.4	104.3
12.4	215.3 ± 20.9	0.227	3.0 ± 1.0	5.3 ± 1.5	109.8
12.5	202.9 ± 29.8	0.215	2.2 ± 0.7	5.4 ± 1.2	87.3
12.6	213.3 ± 22.0	0.23	2.2 ± 0.4	5.4 ± 1.4	101.2
12.7	199.6 ± 16.6	0.232	2.7 ± .04	5.1 ± 1.5	108.7
12.8	203.3 ± 20.8	0.233	2.4 ± 0.5	5.4 ± 1.4	87.4
12.9	209.3 ± 18.4	0.234	2.1 ± 0.3	5.3 ± 1.4	97.4
13.0	196.4 ± 17.3	0.215	2.0 ± 0.0	5.2 ± 1.4	90.5
13.1	210.7 ± 15.6	0.257	2.4 ± 1.1	5.2 ± 1.4	95.0
13.2	213.6 ± 15.7	0.229	2.4 ± 0.5	5.1 ± 1.5	109.2
13.3	201.9 ± 15.0	0.215	2.5 ± 0.5	5.0 ± 1.5	100.1
13.4	197.1 ± 23.9	0.227	2.2 ± 0.4	5.0 ± 1.6	112.1
13.5	208.1 ± 19.9	0.245	2.4 ± 0.5	5.1 ± 1.4	120.5
13.6	206.9 ± 15.5	0.266	3.2 ± 0.8	5.0 ± 1.5	108.9
13.7	194.4 ± 11.7	0.225	2.4 ± 0.5	5.0 ± 1.5	72.5
13.8	185.3 ± 14.3	0.236	2.4 ± 0.5	5.0 ± 1.6	115.4
13.9	193.7 ± 18.5	0.248	2.5 ± 0.5	5.0 ± 1.5	112.0
14.0	188.4 ± 22.2	0.261	2.7 ± 0.5	5.0 ± 1.5	120.4

HLBr (required hydrophile-lipophile balance); SD (standard deviation).

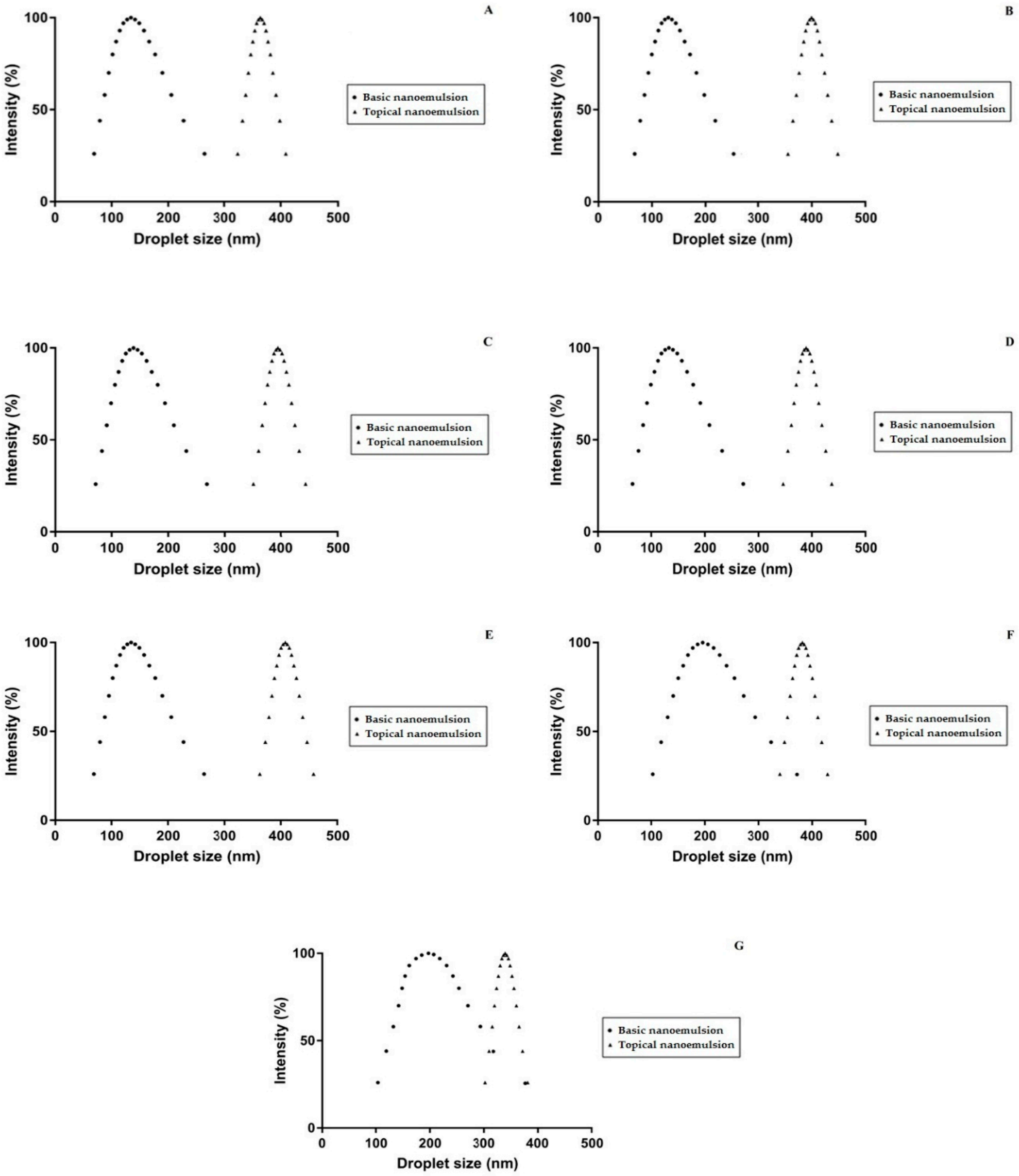

Figure S1. Dynamic Light Scattering (DLS) droplet size distribution of basic and topical nanoemulsion on stability study. (**A**) Day 0; (**B**) Day 1; (**C**) Day 7; (**D**) Day 15; (**E**) Day 30; (**F**) Day 60; (**G**) Day 90. ●: Basic nanoemulsion; ▲: Topical nanoemulsion.
